# Control and Surveillance Operations to Prevent Chronic Wasting Disease Establishment in Free-Ranging White-Tailed Deer in Québec, Canada

**DOI:** 10.3390/ani10020283

**Published:** 2020-02-12

**Authors:** Marianne Gagnier, Isabelle Laurion, Anthony J. DeNicola

**Affiliations:** 1 Ministère des Forêts, de la Faune et des Parcs, 880 chemin Ste-Foy, Quebec City, QC G1S 4X4, Canada; Marianne.Gagnier@mffp.gouv.qc.ca; 2 White Buffalo Inc., 26 Davison Road, Moodus, CT 06469, USA; tony.denicola@whitebuffaloinc.org

**Keywords:** cervids, cervid prions, chronic wasting disease, chronic wasting disease response, culling, infectious diseases, prion containment, prion diseases, white-tailed deer

## Abstract

**Simple Summary:**

Chronic wasting disease (CWD) is a transmissible and deadly disease affecting free-ranging and farmed cervids; no treatment or vaccine is available at this time to cure or prevent CWD. When established in free-ranging cervid populations, CWD is currently impossible to eradicate and leads to potentially irreversible population declines. The first cases of CWD in Québec were detected in 2018 on a red deer (*Cervus elaphus*) farm. Immediately following detection, intensive culling efforts were conducted in a control area around the infected farm to (1) eliminate free-ranging deer that may have come in contact with infected deer from the farm, and (2) to decrease free-ranging deer densities to reduce potential contact between the animals and therefore lower the risk of transmission. To prevent the spread of CWD, we applied legal restrictions regarding the movement of specific anatomical parts of cervids harvested near the affected farm. To determine if CWD was present in free-ranging cervids, we tested 447 white-tailed deer (*Odocoileus virginianus*) harvested through sport hunting in the surveillance zone, 534 white-tailed deer culled from the control area, and 2584 white-tailed deer harvested outside the enhanced surveillance zone and control area. No positive CWD cases were found, suggesting that if the disease is present in free-ranging animals, infection rates are low, and it may still be possible to prevent its establishment in Québec.

**Abstract:**

Chronic wasting disease (CWD), a degenerative and fatal prion disease affecting cervids, was detected for the first time in the province of Québec, Canada, in a red deer (*Cervus elaphus*) farm in the Laurentides region on 10 September 2018. To assess CWD prevalence and control the disease in the free-ranging white-tailed deer (*Odocoileus virginianus*) population, a response plan including enhanced surveillance, population control, regulatory measures, and public outreach was deployed by the Ministry of Forests, Wildlife, and Parks (MFFP). In the 401 km^2^ white-tailed deer control area, a total of 750 free-ranging white-tailed deer were culled over 70 days, from 22 September to 15 December 2018. Of the culled deer, 534 were tested for CWD. We also tested for CWD a total of 447 white-tailed deer hunted from the enhanced surveillance zone and 2584 free-ranging white-tailed deer harvested outside this zone. Regulations were applied to prevent the spread of the disease through movements of infected animals harvested by hunters. Although no case of CWD was detected in free-ranging cervids in Québec in 2018, this does not confirm the absence of the disease in these populations. However, the results suggest that if CWD is present, few free-ranging cervids are infected, making it possible to prevent its establishment in the province of Québec.

## 1. Introduction

Chronic wasting disease (CWD) is a transmissible degenerative and fatal prion disease affecting free-ranging and captive cervids. Once the disease is introduced, the only known effective option to prevent CWD from becoming established in free-ranging cervid populations is to sufficiently reduce densities in the area where the disease has been detected [1,2]. This will decrease contact rates and remove as many potentially infected individuals as possible. It is theorized that low densities limit transmission if contaminated individuals are present [3,4]. The theoretical deer population reduction target density to prevent CWD spread is 1 ≤ deer/km^2^ [5].

Chronic wasting disease was detected in a red deer (*Cervus elaphus*) farm in the Laurentides region, Québec, Canada, on 10 September 2018, the first identified case in the province. All captive animals, a total of 2789 deer, were slaughtered between 13 September and 18 December 2018. Animals 12 months and older were tested (*n* = 1783), and ten additional cases were found, leading to an estimated prevalence of 0.6% (Canadian Food Inspection Agency (CFIA), unpublished data). Despite the investigation conducted, the origin of the farm contamination has not been identified. The Federal government (CFIA) was the lead agency associated with interventions and assessment related to the infected deer farm.

In Canada, white-tailed deer (*Odocoileus virginianus*) management is a provincial responsibility. Ministry of Forests, Wildlife, and Parks (MFFP) of Québec develops and implements a free-ranging white-tailed deer management plan focused on the enhancement of this resource. Issues such as cohabitation with humans, management through hunting, habitat sustainability, maintaining healthy populations, and disease response are undertaken by MFFP. As soon as the first CWD case was confirmed and its exact location identified, the Québec government, through MFFP, quickly deployed a response plan to control the disease, prevent its establishment, and assess its prevalence and distribution in the local free-ranging white-tailed deer population.

In developing its response plan, MFFP considered the following factors: (1) CWD surveillance has never been conducted in the free-ranging deer population of this region, (2) CFIA’s investigation determined that CWD was present in the farm herd for a minimum of 1.5 years prior to detection, and (3) contacts between farmed deer and free-ranging deer along the facility fences have been regularly documented by farm staff and local residents. Those factors led to the premise that CWD transmission into the free-ranging white-tailed deer population frequenting the surroundings of the farm sites was likely. Consequently, the plan objectives and its components were (1) enhanced surveillance to assess CWD prevalence and potentially define the extent of the infected area, (2) control potential CWD transmission and spread through significant and unselective free-ranging white-tailed deer population reduction via culling, (3) prevent further CWD spread through regulation, and (4) inform the public about the regulations and the ongoing actions to prevent CWD establishment through public outreach initiatives.

This plan was developed on the basis of best management practices from other jurisdictions, CWD expert recommendations, recent scientific literature, and white-tailed deer ecology in Québec. Operational and strategic components of the MFFP plan were inspired by the successful New York State CWD response and the recent Norwegian CWD control operation. Moreover, we also considered recommendations to pursue an aggressive population reduction goal made by jurisdictions where CWD has been established despite significant control and eradication efforts.

White-tailed deer in Québec exhibit winter yarding behavior, which was strongly considered during the development of the response plan. In the northern part of their range, from approximately mid-December to mid-April, white-tailed deer leave their summer habitat to congregate in wintering areas [6]. This yarding behavior leads to the concentration of deer from different areas and could rapidly spread CWD to new territories upon return to their respective summer ranges [7,8]. Therefore, we also conducted a preliminary global positioning system (GPS) collaring initiative to complement the population reduction operation and gain a better understanding of the seasonal movement behavior of local white-tailed deer. We sought to get real-time yarding movement data from the local free-ranging white-tailed deer found proximate to the infected farm site to spatially adjust our population reduction operation in case of CWD detection in the free-ranging population.

The primary objective of this paper was to share the experience behind the development and implementation of a CWD response plan. Highlighting the successes and addressing the constraints and challenges we faced in the field related to the rapid deployment of the response plan will benefit other jurisdictions, permitting them to react promptly and adequately should a CWD control response be required.

## 2. Materials and Methods

### 2.1. Surveillance

**Figure 1:**
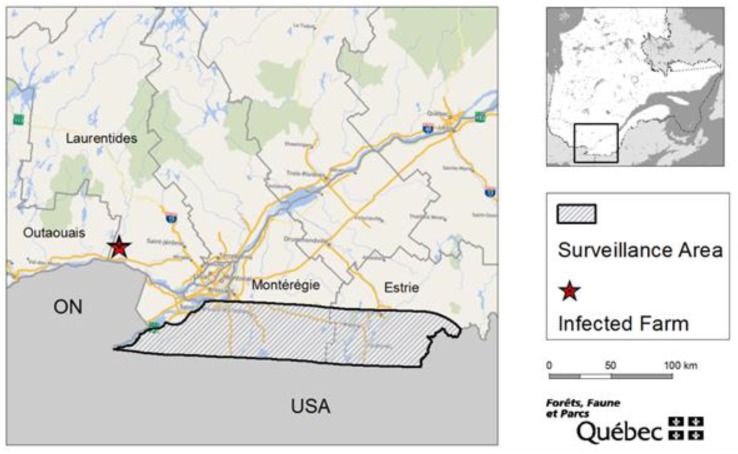
Location of the 2007–2017 chronic wasting disease (CWD) surveillance area and of the infected farm site in 2018.

Starting in 2007, the MFFP implemented a CWD surveillance program in free-ranging white-tailed deer populations from the Estrie and Montérégie regions in southern Québec. The areas targeted by this program are located approximately 70 km from the 2018 CWD-infected farm and are separated from it by the St. Lawrence and the Ottawa rivers (Figure 1). Those regions in the province were considered to have the highest CWD introduction risk in free-ranging deer on the basis of the most recent CWD case detection in a neighboring jurisdiction (NY, USA, 2005) and the high free-ranging white-tailed deer densities. Chronic wasting disease samples came from deer killed by vehicle collisions, as well as from white-tailed deer harvested by hunters and collected through a butcher shop network.

**Figure 2:**
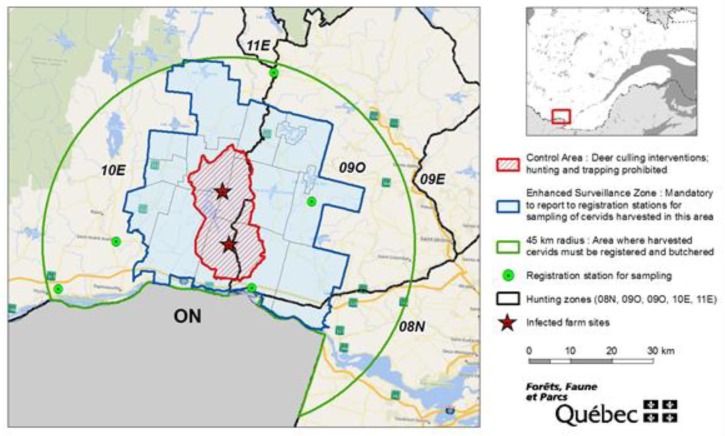
Areas designated with specific measures to control CWD in Québec.

Following the detection of the CWD cases in 2018, in a region where CWD surveillance in free-ranging white-tailed deer had never been conducted previously, an enhanced surveillance zone (ESZ) was established around a free-ranging white-tailed deer control area. The ESZ was a 7.5 km buffer zone that encompassed 15 municipalities, entirely or partially. All hunters who harvested a white-tailed deer or a moose (*Alces americanus*) in the ESZ had to register their animal at one of the five designated wildlife registration stations located within the 45 km radius of the infected farm sites (Figure 2). Throughout the hunting season (i.e., September until late November), MFFP wildlife technicians and biologists were present at the stations to collect samples (obex and retropharyngeal lymph nodes) for CWD testing. Results were available within 2 to 5 business days and were communicated directly to the hunter by phone. Ministry of Forests, Wildlife, and Parks personnel posted at the five registration stations in the ESZ also opportunistically sampled cervids harvested by hunters outside the ESZ that were registered at those stations.

Outside the ESZ, the participation of several butcher shops in other regions of the province in 2018 allowed for a significant expansion of the initial CWD surveillance program, which started in 2007. The network increased from 8 butcher shops in 2 regions to more than 40 butcher shops in 7 regions.

Butcher shops located outside the ESZ did not have CWD-specific safety protocols to follow when handling and processing cervid carcasses. The provincial Ministry of Agriculture, Fisheries and Food (MAPAQ) regulation related to food safety continued to apply. Within the ESZ, CWD results were released to hunters before butcher shops started processing the carcasses. Provincial food safety regulation also applied.

### 2.2. Regulations to Prevent Disease Spread

To prevent the spread of the disease to other regions of the province through the movement of infected animals harvested by hunters near the infected farm, the MFFP applied a regulation adopted in 2012 [9]. This regulation states that certain anatomical parts of a white-tailed deer, moose, or farm cervid carcass culled within a 45 km radius of a location at which a case of CWD has been discovered must remain within that 45 km radius and within the hunting zone in which the animal was harvested. Those parts are:

- spine and spinal cord;
- head, more specifically any part of the brain, the eyes, the retropharyngeal lymph nodes, and the tonsils;
- internal organs (including the liver, the heart, the kidneys, the bladder);
- testicles.

The meat, antlers without velvet, and other unspecified anatomical parts can be moved without restriction.

To prevent the spread of CWD through the movement of captive cervids, MFFP regulations also banned the movement of any cervid kept in captivity within a 100 km radius of infected farm sites, unless transport is to a slaughterhouse. Some cervid facilities within the province, located outside this perimeter, have been placed under quarantine by MAPAQ, given some known epidemiological links with the infected farm.

In 2018, the 45 km and 100 km radii encompassed territories managed by other jurisdictions, where their own regulations applied. Close collaboration and regular information sharing were maintained as the surveillance and culling operations were ongoing.

### 2.3. Population Reduction

#### 2.3.1. Study Area

The private deer farm where the initial CWD-infected red deer was identified was composed of two different sites located ≈12.5 km apart in the Laurentides region and bordered by the Outaouais region westward. Both sites were considered infected due to the documented material and staff movements between the locations. The site where 11 CWD cases were detected was located ≈14.5 km northwest of the village of Grenville, Québec, along the Rivière Rouge, and housed 2224 deer in an approximate area of 1.6 km^2^. The second site, covering an area of ≈0.8 km^2^ and containing 565 deer, was located ≈5.5 km southeast of the village of Boileau, Québec. The area surrounding the two sites was mainly comprised of rolling conifer and hardwood forests intermixed with agricultural fields, wetlands, and low-density housing. Deer density in the area was estimated at 2–3 deer/km², with an uneven deer distribution in the landscape. Hunting in the region was focused on the harvest of adult males, resulting in a higher proportion of females than males in the local white-tailed deer population.

#### 2.3.2. Methods

The white-tailed deer population reduction zone was established on 19 September 2018 using a 7.5 km radius around each site, resulting in a 401 km^2^ control area (Figure 1). This distance was determined on the basis of other jurisdictions’ experiences in deer population control related to CWD, on estimated deer density, and estimated home range. Moreover, logistical and technical feasibility to deploy a major and complex population reduction operation in this territory was also taken into consideration in the area’s size determination. The legal delineation of the control area was identified according to existing physical and territorial boundaries.

To optimize culling efforts and public safety, we prohibited sport hunting and trapping in the control area during the traditional season from 21 September to 18 November 2018. In the field, MFFP’s wildlife officers addressed safety concerns and ensured law enforcement presence to reduce public interference with the population reduction operation, to reassure local residents, and to prevent CWD movement through infected animals or parts.

A field coordination headquarters was established in the control area for the duration of the population reduction operation. This site included an office, a sampling lab, equipment storage facilities, and a freezer trailer used for deer carcass storage. The headquarters was mainly used as a meeting place for the culling teams, a field communication and coordination center, a primary data collecting and processing office, and a sample preparation and shipping site.

Ministry of Forests, Wildlife, and Parks hired an organization that specialized in white-tailed deer management to help facilitate the deer population reduction operations. They advised and trained MFFP’s personnel regarding various aspects of the operations (i.e., technical and strategic). Specifically, information and training were provided regarding firearm selection, deer culling methodologies, bait site distribution and selection, data organization and management, and deer culling implementation strategies.

All experimental operation related to population control was authorized through a Wildlife Management Permit ("permis de gestion de la faune" (SEG)), delivered by the Ministry of Wildlife, Forest and Parks, Québec, Canada, permit #2018-09-19-076-15-G-F.

#### 2.3.3. Deer Culling

Intensive culling efforts of free-ranging white-tailed deer were conducted from 22 September to 15 December 2018. The population reduction operations ceased at the estimated time of the year when deer traditionally move to their winter habitat. Observed and documented white-tailed deer winter yarding movements in the southern part of the zone resulted in local targeted deer leaving the control area. In contrast, in the northern part of the control area, untargeted deer from outside the control area were massively migrating inside (MFFP, unpublished data). Deer population reduction efforts focused on reducing deer densities as low as possible within the defined area to (1) eliminate deer that could have potentially been in contact with a CWD-infected red deer or with infected material from the farm, and (2) reduce the probability of potential CWD transmission between free-ranging deer if the disease had been transmitted outside of the farm. All encountered free-ranging white-tailed deer were culled, including fawns, without any sex or age discrimination.

Two culling approaches were used to conduct population reduction operations: mobile shooting and static shooting. An opportunistic mobile shooting strategy, on properties where permission had been granted, was applied from the start of the culling operation, because deer attraction to bait was limited due to the abundance of natural food sources. Typical mobile operations consisted of two teams of three persons operating within the control area from approximately 5:00 pm until 2:00 am. Night shooting was preferred to daytime shooting as deer were calmer and less easily disturbed; more actively feeding in open, accessible areas; and human activity was minimal. Daily planning ensured that team coverage did not overlap to maintain efficiency in covering as much of the control area as possible on a nightly basis. All mobile shooting tracks were recorded using a mobile phone application (Track Kit 2.9.1, https://track-kit.net/) and compiled to assess total effort and area covered throughout the project. The list of landowners that provided permission to access their property for baiting and culling were updated daily and integrated into moving map GIS software (ArcPad 10.4.1, ArcMap 10.2, ESRI, Redlands, CA, USA). Deer were approached using a vehicle and a spotlight and shot at a distance of 10–175 m, using a bolt action 223 caliber scoped rifle with a mounted bipod. The vehicle was fitted with a platform to ensure benchrest-level shooting precision in all directions (360°). Cartridges were loaded with highly frangible 50 gr projectiles (muzzle energy ≈1500 J; Hornady V-max, Grand Island, NE, USA). Accuracy and precision of firearms (<1 cm five-shot groups at 100 m) was tested on a firearm range prior to shooting operations, and a rangefinder was used to determine distances during shooting operations. The point of aim selected for this project was the upper cervical vertebrae to ensure an instantaneous death [10]. This shot placement also resulted minimal blood and tissue loss in the field, to limit possible CWD environmental contamination, and the preservation of select tissue samples. Deer were primarily shot while foraging in agricultural fields. Immediately after shooting, entire carcasses were collected and brought to the operational headquarters for sampling and disposal.

Deer were also shot from static shooting locations over bait from a tree stand, existing permanent hunting blinds, or from a stationary vehicle. Preparation for static shooting operations began in late-October with the strategic placement of bait on properties with granted permission and suitable locations to cull deer. Bait consisted of whole corn, but we added apples and carrots on some bait sites, and transitioned to whole corn on all sites as freezing conditions persisted. Each bait site was monitored with an infrared trail camera (Spypoint, Force-S, Victoriaville, QC, Canada; Ridgetec, Summit-4, Lethbridge, AL, Canada; Moultrie, M-80XT, Birmingham, AL, USA; Moultrie, M-999i, Birmingham, AL, USA; Reconyx, PC800, Holmen, WI, USA) to document deer activity. The sites were baited daily at a consistent time, and camera data were collected to identify deer movement patterns. Once bait acceptance was high, and a suitable pattern was identified, a shooter was stationed at the site from approximately 3:00 pm until 9:00 pm to remove the associated deer.

We maintained a high concentration of cameras over baited areas proximate to the infected farm site during culling operations that allowed us to identify individuals in the area. We then accounted for each deer, or group, as they were culled, or became absent when winter weather induced emigration from the area.

### 2.4. CWD Sampling and Testing

At the beginning of the population reduction operation, the level of free-ranging deer infection rates and contamination in the surroundings of the infected farm were not known. As a precaution, measures were taken when handling and transporting the culled deer carcasses to avoid potential contamination of the natural environment through contaminated fluids. They were handled and processed at a temporary field lab for CWD tissue sampling with special care paid to avoid any fluid contamination of the site and material. Subsequently, all carcasses were transported and disposed through incineration by a specialized company.

All adult deer culled in the control area were sampled (i.e., obex and retropharyngeal lymph nodes) and analyzed by the MAPAQ using an enzyme-linked immunosorbent assay (ELISA). No samples were taken from fawns, as tests are not reliable for detecting CWD in animals that have been infected for less than 12 months [11]. Sampling was done the night of harvest, at the temporary field lab, by a team of two to three wildlife technicians and biologists. This team also handled carcass disposal in a freezer trailer on site for periodic pick-up and incineration. Lab shipments occurred the next morning, by an express daily courier service, and test results were available that same day.

We used the 2018 free-ranging white-tailed deer cull data to conduct an approximation to the hypergeometric distribution [12] to estimate CWD prevalence in the free-ranging white-tailed deer population surrounding the infected farm site. A CWD test sensitivity of 80% was applied [13] in order to be conservative and considering the test inefficiency to detect recently infected animals.

### 2.5. Capture and GPS Collar Deployment

Capture activities were conducted from 16 to 21 November 2018. The capture area was determined considering the following factors: (1) location of the CWD cases found on the farm (affected enclosures location), (2) documented free-ranging white-tailed deer habitat used and location in the vicinity of the affected enclosures (through cameras and tracks), and (3) the availability of remaining individuals in the area (camera data) when collaring operation began.

We approached white-tailed deer in a vehicle during the late evening and early morning hours at bait sites placed along public roadways and private roadways/properties where permission was granted. Capture activities occurred in a neighborhood along the northern boundary of the CWD-infected farm site. Animals were immobilized via remote drug delivery using carbon dioxide-powered projectors (G2 X-Caliber, Pneu-Dart, Inc., Williamsport, PA, USA). Darts of 2 ml in length were used to administer a combination of 4.4 mg/kg Telazol (Fort Dodge Animal Health, Fort Dodge, IA, USA) and 2.2 mg/kg xylazine hydrochloride (Anased®, Akorn Animal Health, Lake Forest, IL, USA; 100 mg/mL). Darts also contained a transmitter to aid in locating the sedated animal. After darting an animal, the handler waited 10–15 min before locating the deer via radio-telemetry or through direct observation. Ophthalmic ointment (Puralube, Pharmaderm, Melville, NY, USA) was applied to prevent ocular desiccation, and masks were placed over the eyes. All captured deer were fitted with a single button-style ear tag to aid in individual identification and also included a phone number and information on human consumption. Data collected for each animal handled included estimated weight based on chest circumference [14], age based on tooth wear and replacement [15], GPS location of capture, and general health observations. Each deer was fitted with a global positioning system (GPS) collar, with very high frequency (VHF) capabilities (Vectronics Vertex Survey GPS collar, Vectronics Aerospace, Berlin, Germany). We programmed collars to acquire fixes twice a day. Deer were positioned either sternally or on their left side while the devices were affixed. All deer were released near capture locations, in areas with limited exposure to potential hazards (i.e., roads, water features, human disturbance). Prior to release, deer received 0.26 mg/kg intramuscular of the reversal agent atipamezole HCl (Revertor®, Modern Veterinary Therapeutics, LLC, Miami, Florida, USA; 5 mg/mL) and were monitored until ambulatory.

### 2.6. Public Outreach

A website was created to inform the public about the ongoing enhanced surveillance and population reduction operations during fall 2018 and to share regulations and measures associated with the recent CWD response plan. This website displayed an interactive map and a “frequently asked questions” section, which was updated regularly to inform and engage the public. Three informational press releases were issued, and 19 informational posts were published on the MFFP Facebook page. The MFFP provided 125 interviews and appeared on two specialized television programs. Furthermore, four public assemblies were held for the residents living in the control area, each engaging around 70 to 170 individuals.

In the control area, landowners were met by MFFP representatives and wildlife officers to explain the response plan objectives, to inform them about CWD and its potential impacts on the free-ranging deer population, as well as to obtain their permission to conduct the deer cull on their property. Throughout the population reduction operation, MFFP maintained regular contact with local municipal officials to keep them informed about the progress of the response plan. From the start, their support was considered essential to inform local residents as well as to obtain public endorsement toward the response plan actions.

In the enhanced surveillance zone, MFFP staff posted at the registration stations also provided information to hunters and to the public about the response plan goals, CWD epidemiology and impacts, and the function of the enhanced surveillance through which hunted deer were tested.

Finally, many documents were produced and released, including brochures, public notices, and posters that provided information about CWD, the ongoing operations, as well as related regulations.

## 3. Results

This section presents more specific data related to the planning components from which results were measurable.

### 3.1. Population Reduction

We culled 750 deer during 70 days of fieldwork—415 during mobile operations and 335 during static operations. Harvest per day ranged from 2 to 31 deer. Only 2 days of removal activity were canceled due to inclement weather, and 13 days were redirected to administrative responsibilities during the tenure of the plan. Throughout the culling operations, 6519 km was traveled in search of deer during mobile sharpshooting, resulting in an average encounter rate of 1 deer observed per 5.9 km traveled. In addition to the culling efforts, 24 deer and 1 moose were sampled from the control area, all related to reported animals that were found dead (i.e., vehicle collision, natural mortality, and seized animals).

### 3.2. CWD Sampling and Testing

Prior to the 2018 CWD detection in Québec, the surveillance program conducted between 2007 and 2017 in Montérégie and Estrie regions led to the testing of 9300 free-ranging white-tailed deer, and no cases of CWD were detected.

After 2018 CWD cases were detected in a red deer farm of the Laurentides region, we analyzed samples from 447 deer and 21 moose harvested by hunters from the ESZ, and all came back negative for CWD. No CWD surveillance efforts had been conducted in this region before 2018. In addition, MFFP personnel posted in the 5 registration stations located in the ESZ sampled 606 white-tailed deer and 11 moose harvested by hunters outside the ESZ, and none tested CWD-positive.

During the 2018 population reduction operations, we submitted 534 samples from deer culled in the control area, and all tested negative for CWD. We culled 216 fawns during the operation; however, they were not sampled, as tests are not reliable for animals infected for less than 12 months. The 24 deer and the moose found dead in the control area also tested negative for CWD.

Through the expanded butcher shops network outside the ESZ, we collected 1978 additional free-ranging white-tailed deer samples throughout the province in 2018, and none tested positive for CWD.

To assess CWD presence in the control area, 534 white-tailed deer were tested, within a population evaluated at 800–1200 individuals (2–3 deer/km^2^ in a zone of 401 km^2^). Our calculations led to a local CWD prevalence currently estimated to be <1%, with a 99% probability of detection. Although no CWD cases were detected through 2018 sampling, which was considered in the calculations, this represents the estimated prevalence if CWD had spread into the free-ranging white-tailed deer population of the area.

### 3.3. Capture and GPS Collar Deployment

**Figure 3:**
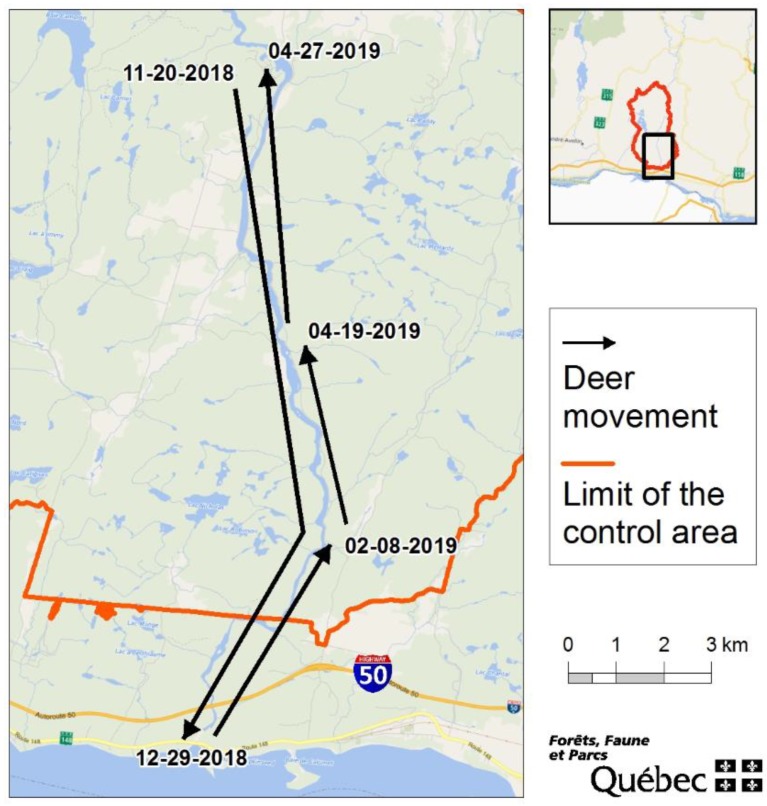
Movements of the female collared deer between summer and winter habitats, from 20 November 2018 to 27 April 2019.

One adult female white-tailed deer and one male fawn were captured and collared. The male fawn remained in the area of its capture. Conversely, the adult female moved ≈14 km south of its capture location in mid-January 2019. This individual migrated to a known and documented wintering area south of Highway 50 (Figure 3), outside of the control area.

The two collared deer were culled in May 2019. At that time, they had returned to the location where they were captured. Both specimens were sampled, tested, and results came back negative for CWD.

## 4. Discussion

The population reduction and enhanced surveillance operation conducted by the MFFP in 2018 was implemented rapidly and efficiently, considering the complexity and the magnitude of deploying such a plan. Intensive operational and logistical efforts allowed us to begin the free-ranging deer cull only 12 days following the first CWD case detection in the province. The use of MFFP’s existing network and experience related to deer management and wildlife disease control ensured a rapid project initiation. Enhanced surveillance, population control, and regulatory measures were implemented promptly, fostering a reduction of the risk of transmission and consequently of the probability of CWD establishment in the area. In addition, the rapid rollout of the CWD operation included many communication efforts (i.e., press release, public assembly, MFFP website updates, MFFP Facebook page publication, individual meetings with landowners, etc.). This compressed timeline and the evolving messaging made internal and external communication activities challenging. This was especially difficult when sharing up-to-date information about the response plan and its goals while ensuring comprehension by the public.

In the 401 km^2^ control area, given the initial free-ranging deer estimated density (800–1200 deer) and the total number of deer culled (*n* = 750), we determined that our goal of reducing the free-ranging white-tailed deer population had been reached. Moreover, the relative hunting success documented in the control area during the 2019 season was lower than those registered prior to 2018 (MFFP, unpublished data), which corroborates our 2018 population reduction conclusions. Deer densities in the areas proximate to both red deer farm sites were decreased significantly, but it was difficult to quantify the densities at the conclusion of shooting operations because seasonal movements had begun. The seasonal influx of deer into the Boileau area (northern farm site) in late-November made it impossible to delineate local deer from seasonal immigrants. The area is a documented deer winter yard (MFFP, unpublished data), and early winter weather resulted in seasonal immigration into the area before the conclusion of shooting operations. During and after culling operations, camera data and field observations allowed us to estimate that <10 known individuals remained in the 1.5 km buffer zone around the southern site of the farm, where all CWD cases were found.

Chronic wasting disease testing conducted during the population reduction efforts suggest that if CWD is present in the local free-ranging white-tailed deer population of the control area, then local prevalence was estimated to be <1%. This is encouraging, but more time is needed until disease absence is confirmed given the recent timeline of disease detection. This is of particular concern because CWD tests are not usually effective for disease detection in recently infected animals, and thus our data may not have been fully representative of infection rates.

The collection of GPS data for movement analysis was added later in the operation. As a result, densities were already low, and few animals remained available for capture. The movement data from one individual reflected the potential transmission risk posed by migratory behavior and excursions. The collared male fawn remained in the area of its capture, partly included in a known winter deer yard (MFFP, unpublished data). Thus, this lack of movement was not unexpected. Moreover, the fawn’s social group was harvested, and thus it was not exposed to learned winter yarding behavior. The female collared deer provided insights related to distance and direction traveled to wintering areas used by individuals living proximate to the CWD-positive farm site. It confirmed and supported the local white-tailed deer winter movement behavior estimated by MFFP local deer management specialists that deer from this area often travel ≥10 km to reach an important and well-documented winter yard located south of the control area (MFFP, unpublished data). Unfortunately, logistical constraints, difficult weather conditions, and timing impacted the ability to capture more deer proximate to the CWD-infected farm site. Therefore, we lost the opportunity to better understand broader migratory behavior and whether the collared female was an anomaly or representative of deer migration behavior in the area. Regardless, her movements are a stark reminder of how a single individual can expose a distant population to CWD as a result of seasonal habitat shifts.

Although the plan implementation overall was a success, challenges related to human dimensions (e.g., citizen understanding and acceptance of plan objectives) were faced as the operation progressed. This was one of the most difficult aspects of the 2018 CWD response plan. Lack of support for the population reduction operation by local hunters and landowners negatively impacted our ability to fully achieve the initial objectives of the response plan. Population reduction actions needed to be implemented rapidly, and this partially prevented the adequate establishment of public understanding and awareness. In addition, because no positive CWD cases were found in the free-ranging white-tailed deer population, it was difficult for the public to understand the need to continue deer population reduction in the control area. As a result, public endorsement gradually decreased as the free-ranging white-tailed deer population reduction operation continued. Overall, the urgency to take action to prevent CWD establishment in the free-ranging white-tailed deer population and the potential impact of this disease at a provincial level were often underestimated by relevant local groups and individuals. Tremendous efforts were deployed locally by MFFP representatives to reach a high level of understanding by local population and municipal officials. The educational efforts focused on CWD epidemiology and mechanisms, potential impacts on the free-ranging deer population, and the cost-efficiency and necessity of such a response plan. However, given the observed decrease of local support, it is likely that the messaging and the tools used did not fully succeed in convincing targeted groups of the importance of achieving the response plan objectives.

Public understanding and acceptance of CWD management in Québec will remain challenging in the years to come, especially if CWD is detected in another area. The authorities responsible for provincial deer management will have to increase their efforts and use creativity in their communications in order to gain greater public support. In an era where citizens are submerged with information, it remains difficult to capture the public interest when the threat does not seem imminent. This is not unique to CWD, given that the MFFP faced similar challenges communicating with the public regarding other wildlife management issues (e.g., raccoon rabies, invasive species, etc.).

## 5. Conclusions

Overall, the 2018 Québec CWD response plan deployment was a success, resulting in a significant reduction in the risk of CWD transmission and establishment. In addition, we estimated a very low CWD prevalence, if it was the case that the disease had spread from the infected farm into the local free-ranging white-tailed deer population.

Although no case of CWD was detected in free-ranging cervids in Québec, this does not confirm the absence of the disease in these populations. These conclusions are based on (1) diseases being challenging to detect in free-ranging wildlife populations when infection rates are low, and (2) CWD tests not usually being effective for disease detection in recently infected animals (i.e., <12 months). However, our 2018 results suggest that if CWD is present in Québec, few free-ranging cervids are infected, making it possible to prevent its establishment in the province.

In 2019, the MFFP continued its efforts to (1) maintain an enhanced surveillance zone near the CWD cases found in 2018 to insure early CWD detection, and (2) maintain a low deer density, through sport hunting, in the area where the disease was detected in order to reduce the contact and transmission risk among potentially CWD-infected free-ranging deer.

In the coming years, the MFFP will (1) maintain an enhanced surveillance program in order to detect the emergence of CWD cases in the free-ranging deer population and react rapidly with control actions, and (2) assess the potential for regulatory measures and best management practice improvements to prevent a new introduction of CWD and its transmission.

Despite the fact that rapid deployment of CWD surveillance and control interventions is possible, it is important to keep in mind that they are costly, energy-consuming, and that their success in combating the establishment of the disease is uncertain. Research to find effective prevention and control tools is therefore crucial for the future management of CWD.
